# Atypical pulmonary thromboembolism caused by the mutation site *SERPINC1* of the antithrombin III gene: A case report

**DOI:** 10.1097/MD.0000000000039175

**Published:** 2024-08-02

**Authors:** Miaoyuan Lin, Xishi Sun, Jun Wu

**Affiliations:** a Affiliated Hospital of Guangdong Medical University, Zhanjiang, China.

**Keywords:** anticoagulant therapy, antithrombin deficiency, pulmonary thromboembolism, *SERPINC1*

## Abstract

**Background:**

Deficiency of natural anticoagulant antithrombin was first reported as a genetic risk factor for venous thromboembolism, antithrombin III (AT III) is encoded by the serpin family C member 1 (*SERPINC1*) gene, consisting of 432 amino acids, including 3 disulfide bonds and 4 possible glycosylation sites. Studies have shown that hereditary AT deficiency increases the incidence of venous thromboembolism by up to 20 times.

**Case presentation:**

The case presented a 27-year-old young man with no acquired risk factors and a sudden onset of right lower extremity venous thrombosis and pulmonary embolism. A heterozygous mutation in gene *SERPINC1* of c.1154-14G>A was detected in the patient, which is a deleterious mutation resulting in reduced AT III activity and increased risk of thrombotic events. The patient received anticoagulant therapy for approximately 5 months, and the thrombus gradually dissolved and no recurrent thrombotic events occurred during follow-up.

**Discussion:**

AT deficiency is a rare autosomal dominant genetic disease, they are mainly divided into 2 types according to the different effects on the structure or function of the encoded protein. The patient had a mutation in the *SERPINC1* gene (c.1154-14G>A). Several cases of this type of mutation have been reported since 1991, and it is classified as AT deficiency type I.

**Conclusion:**

Thrombosis in patients with antithrombin deficiency is often unpredictable and can lead to fatal pulmonary embolism. Early genetic testing for hereditary thrombophilia in venous thromboembolism patients without obvious high-risk factors is critical. Long-term anticoagulation treatment is an effective treatment, for this type of type I AT III deficiency combined with pulmonary embolism patients, warfarin is an effective anticoagulant drug.

## 1. Introduction

Venous thromboembolism (VTE) is a widespread and preventable health concern globally, impacting around 0.1% to 0.2% of individuals each year. Pulmonary embolism, the most severe complication of venous thrombosis, occurs in about one-third of patients and can be life-threatening.^[1]^

Thrombophilia, characterized by a predisposition to blood clot formation due to genetic or acquired factors affecting anticoagulant proteins, clotting factors, or other risk factors, can contribute to the development of VTE. Deficiency of natural anticoagulant antithrombin was first reported as a genetic risk factor for venous thromboembolism. Since then, deficiency of natural anticoagulants such as antithrombin III (AT III), protein C, and protein S has been associated with hereditary venous thrombosis. Acquired thrombophilia can stem from conditions like antiphospholipid syndrome, tumors, surgery, immobilization, advanced age, and oral contraceptive use.^[2–4]^

This article will present a case study of a patient with pulmonary embolism resulting from a mutation in the serpin family C member 1 (*SERPINC1*) gene responsible for AT III, causing type I hereditary AT III deficiency. The patient’s case will be evaluated alongside current clinical guidelines and research to provide insights into the diagnosis and management of similar individuals.

## 2. Materials and methods

### 2.1. Clinical materials

A 27-year-old male patient was admitted to the Department of Critical Care Medicine at the Affiliated Hospital of Guangdong Medical University on August 27, 2021, with complaints of sore throat for 1 week and dizziness for 6 hours. The patient reported experiencing a sore throat with cough and expectoration 1 week prior, and suddenly developed dizziness without an obvious trigger 6 hours before admission. Initial tests revealed elevated troponin I levels (269 ng/L), and the patient was treated symptomatically with nutrition for the heart muscle and antibiotics in the emergency department. Blood pressure was measured as 95/56 mm Hg (1 mm Hg = 0.133 kPa), heart rate as 110 bpm, and the patient was transferred to the critical care unit with a provisional diagnosis of viral myocarditis.

**Figure 1.**
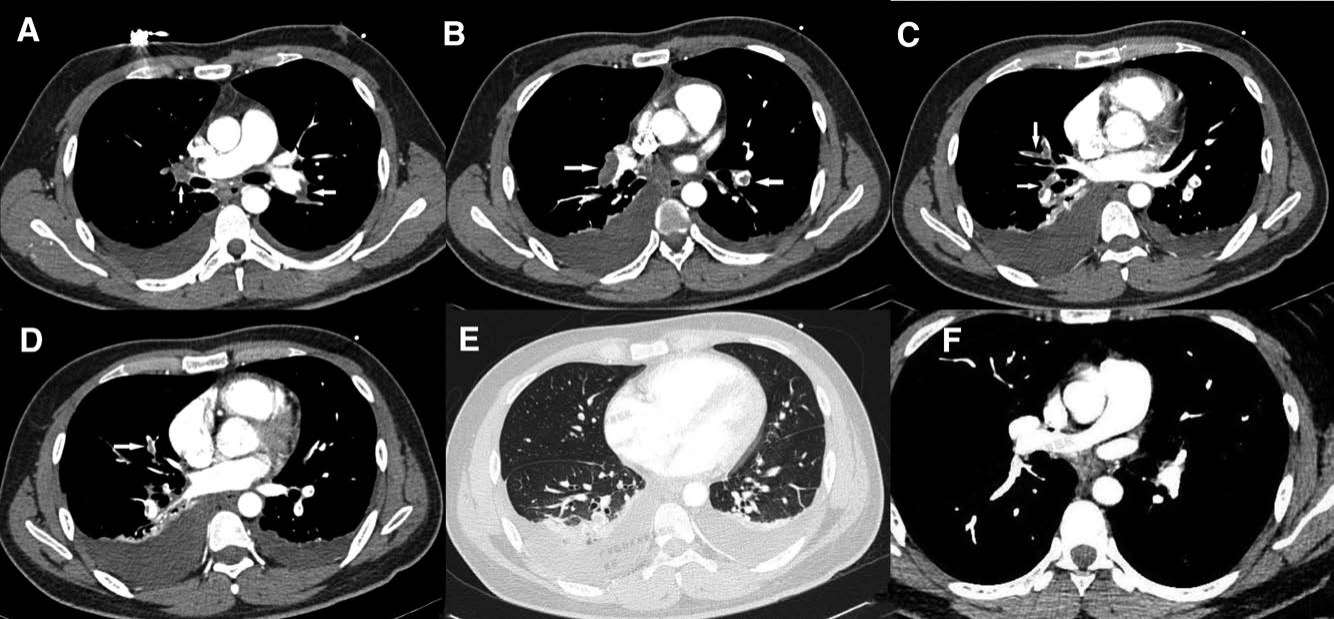
Lung CT during hospitalization and CTPA after discharge (arrow indicates pulmonary filling defect). CT = computed tomography, CTPA = computed tomography pulmonary angiography.

Physical examination: body temperature was 36.5°C, pulse 125 times/min, respiratory rate 31 times/min, blood pressure 108/82 mm Hg, no cyanosis, thick breathing sound in both lungs, no rales or dry rales, no other positive signs. Coagulation function: D-dimer: 17.79 mg/L, fibrin degradation products 78.85 μg/mL; Blood gas analysis: pH value was 7.31, oxygenation index was 217.8, oxygen partial pressure was 98.0 mm Hg; White blood cell count: 15.98 × 10^9^/L; NT-pro-B type natriuretic peptide 2613 pg/mL; cardiac color Doppler ultrasonography showed right ventricular enlargement; the duplex ultrasonography of the lower extremity veins showed the formation of thrombi in the right external iliac vein, common femoral vein, superficial femoral vein, deep femoral vein, popliteal vein, anterior–posterior tibial vein, and peroneal vein. On August 31, 2021, chest computed tomography plain scan + enhanced scan showed filling defects in the distal parts of both pulmonary arteries and in each pulmonary artery branch (Fig. 1A–D), “inflammation” in the lower lobes of both lungs, bilateral pleural effusion, especially on the right side, accompanied by compression atelectasis in the adjacent lower lobes of both lungs (Fig. 1E). Other routine laboratory and biochemical tests showed no abnormalities, and the patient was diagnosed with acute pulmonary embolism (medium to high risk).

After admission to the critical care unit, the patient received subcutaneous injection of bemiparin sodium 2500U once daily from August 28 to August 30, and subcutaneous injection of enoxaparin sodium 6000U every 12 hours from August 30 to August 31, along with other supportive treatments such as stabilizing the myocardium, controlling heart rate, pain relief, bronchodilators, and antibiotics. The patient was transferred to the Department of Respiratory and Critical Care Medicine at Guangdong Medical University Affiliated Hospital on September 1, 2021, once stable. Posttransfer, the patient received nasal oxygen therapy, subcutaneous injection of enoxaparin sodium 7000U every 12 hours, and oral warfarin 3 mg once daily for anticoagulation. Follow-up tests showed white blood cell count at 10.71 × 10^9^/L and C-reactive protein at 30.80 mg/L, with indications of lung inflammation and pleural effusion, suggesting a concomitant lung infection, for which ceftriaxone was added to the treatment regimen. On September 11, 2021, the international normalized ratio (INR) was 1.97, leading to the discontinuation of heparin therapy. Tests for antiphospholipid antibodies and lupus anticoagulants were negative, and protein S and protein C activities were normal. The patient was found to have low AT III activity upon admission. Prior to discharge, repeat tests showed significant decreases in D-dimer, NT-pro-B type natriuretic peptide, and cardiac enzyme levels, along with improvement in symptoms. The patient was discharged on September 13, 2021, with a prescription for warfarin 3 mg once daily for maintenance therapy and scheduled outpatient follow-ups.

**Figure 2.**
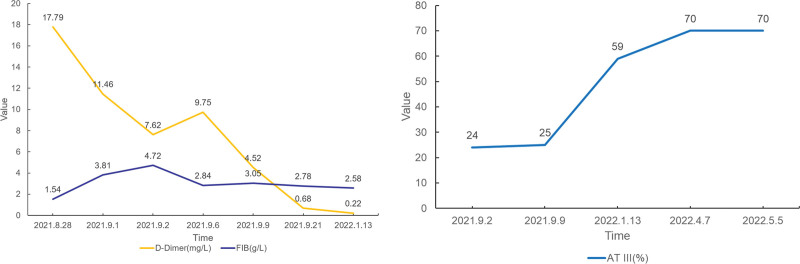
Changes of D-dimer, FIB, and AT III during hospitalization. FIB = fibrinogen.

**Figure 3.**
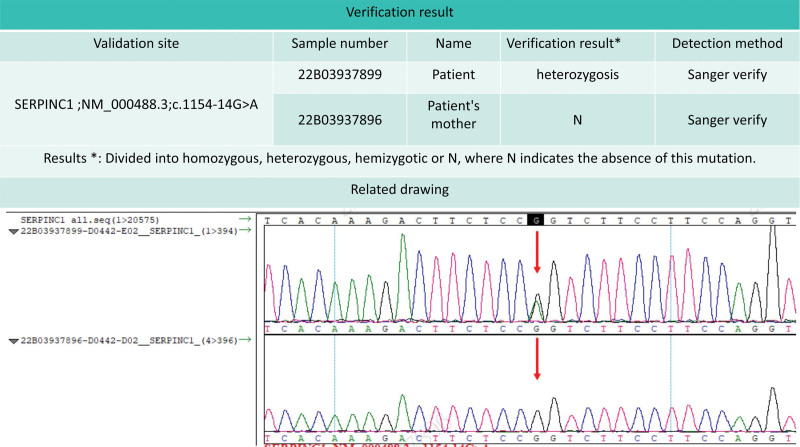
Patient and his mother genetic test verification report.

A follow-up computed tomography pulmonary angiography on January 7, 2022, showed good visualization of the main pulmonary artery, bilateral pulmonary arteries, and their branches without evident filling defects (Fig. 1F). A repeat duplex ultrasonography of the lower extremity veins on February 10, 2022, indicated patency of the right lower extremity vein after treatment, with gradual normalization of fibrinogen and D-dimer levels and a slight increase in AT III levels, albeit remaining below normal values (Fig. 2). Other coagulation parameters, including prothrombin time, activated partial thromboplastin time, and thrombin time, showed slight elevations during hospitalization and follow-up without clinical significance. A family history investigation revealed that the patient’s father, who had passed away due to colon cancer, had a history of lower extremity thrombosis, while the patient’s mother had no detectable mutations upon genetic testing (Fig. 3).

### 2.2. Gene sequencing

Peripheral blood was collected from the patients, and the exon region of about 20,000 genes in the human genome and mitochondrial genome were detected by chip capture high-throughput sequencing (Shenzhen Peking University Medical Laboratory).

### 2.3. Mutation gene evaluation

According to the chief complaints of the patients, the pathogenic genes included in the OMIM database were analyzed, the artificial intelligence software SpliceAI was used for splice site prediction, and the PhyloP Vertebrates and PhyloP Placetal Mammals software were used for nucleic acid conservation prediction. The pathogenicity of the mutation was evaluated according to the ACMG guidelines.

## 3. Result

### 3.1. Gene sequencing

**Figure 4.**
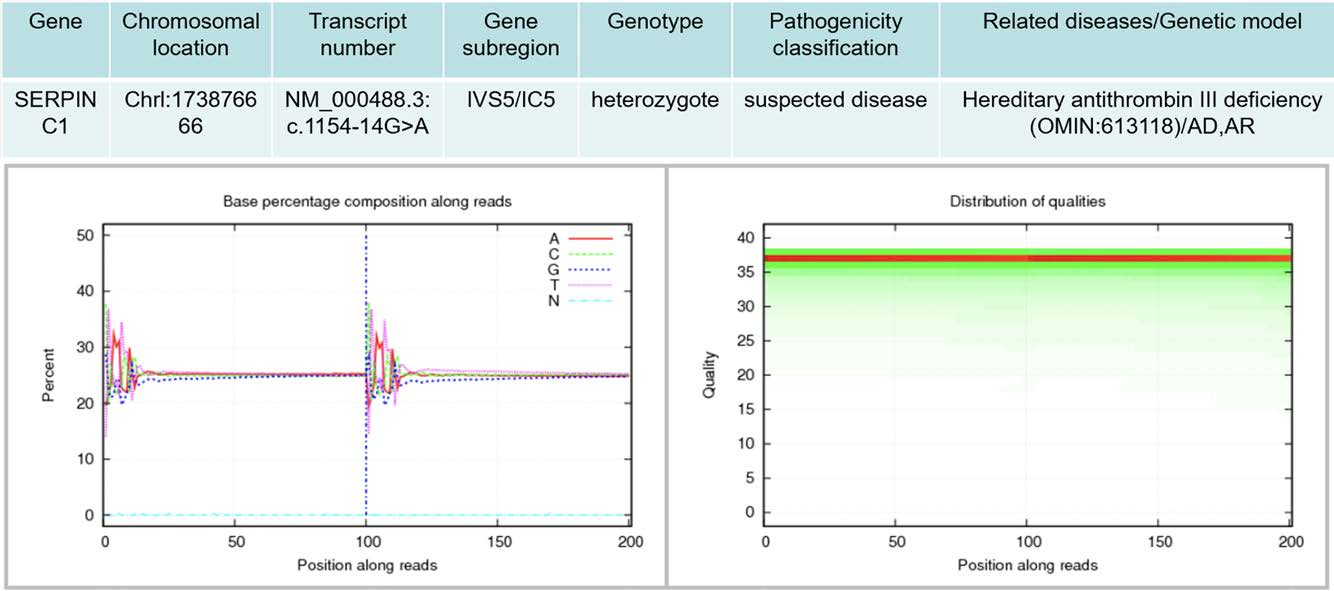
Patient genetic test results.

SERPINC1; NM_000488.3: c.1154-14G>A heterozygous mutation was detected (Fig. 4). There have been reports on the pathogenicity of this variant.^[5–9]^ SpliceAI software predicted that the mutation site was harmful, and PhyloP Vertebrates and PhyloP Placetal Mammals software predicted that the mutation site was not conservative.

### 3.2. Pathogenicity prediction

According to ACMG guidelines, this variant was interpreted as a suspected pathogenic variant, PS4 + PM2 + PP4. The evidence items are as follows: 1PS4: the frequency of the variant in the diseased group was significantly higher than that in the control group. 2PM2: the variant was not found in the normal control population in the ESP database, the 1000-person database, and the EXAC database (or a very low-frequency site in recessive genetic diseases). 3PP4: the phenotype or family history of the variant carrier was highly consistent with a single-gene genetic disease.

## 4. Discussion

AT III is an important blood coagulation inhibitor, which can inactivate coagulation factors. Its main target is thrombin, but it also plays a role by inhibiting coagulation factors such as Xa, IXa, XIa, and XIIa.^[10]^ AT III is encoded by the *SERPINC1* gene, consisting of 432 amino acids, including 3 disulfide bonds and 4 possible glycosylation sites. Studies have shown that hereditary AT deficiency increases the incidence of VTE by up to 20 times.^[11]^ Antithrombin deficiency is a rare autosomal dominant genetic disease, with an incidence of 1/2000 to 3000, caused by heterozygous mutations in the *SERPINC1* gene. Homozygosity is extremely rare, and most patients die before birth, with the AT III level of most patients lower than 50% of the normal value.^[12]^

In patients with thrombophilia, genetic factors account for about 30% to 50%, most of which are caused by mutations in coagulation/anticoagulation factor genes, such as Leiden mutation in factor V gene, G20210A mutation in prothrombin gene, C677T mutation in methylenetetrahydrofolate reductase gene, and defects in protein S, protein C, AT III, and plasminogen activator inhibitor-1.^[13]^ Hereditary AT deficiency was first described by Egeberg in 1965 as a risk factor for thromboembolism.^[14]^ There are many reported variants of the *SERPINC1* gene encoding AT III, a total of 429 mutation sites have been identified in the human gene mutation database. Clinically, they are mainly divided into 2 types according to the different effects on the structure or function of the encoded protein. Type I: classical deficiency, characterized by AT III synthesis disorders in patients, which are manifested as reduced plasma concentration and activity of AT III, so the plasma AT III activity is <50%; Type II: mainly affects the function of the domain binding to thrombin or heparin, but the content of AT III antigen in plasma is normal, but its activity is weakened.^[15]^ The patient had a mutation in the *SERPINC1* gene (c.1154-14G>A). This mutation has been reported since 1991, and it is classified as AT deficiency type I.^[16]^

PE is a common and potentially life-threatening cardiovascular disease. Due to its wide range of manifestations and often nonspecific, it is difficult to diagnose. The patient had a sore throat and dizziness after catching a cold as the main symptoms. Emergency examination showed increased myocardial enzymes, NT-pro-BNP, and infection indicators. The patient was initially diagnosed with viral myocarditis and given symptomatic treatments such as antiinfection and nutritional myocardial therapy, but the efficacy was poor. The patient subsequently had a decrease in blood pressure, shock index >1, and shortness of breath symptoms that gradually aggravated. D-dimer was significantly increased, and multiple venous thrombosis was found in the right lower limbs. Pulmonary embolism was diagnosed by chest computed tomography plain scan + enhancement. After admission, the activity of AT III was significantly decreased several times. The patient was younger, and there were no risk factors such as surgery, trauma, cancer, or special medication history. Combined with the results of gene detection, the diagnosis of hereditary AT III deficiency was considered. Compared with other patients with mild thrombosis, patients with AT III deficiency have a higher risk of venous thromboembolism and a higher recurrence rate. They often show a serious tendency to thrombosis, and even have thrombosis in childhood.^[9]^ The patient was an accountant and was sedentary. It is speculated that the blood reflux of the lower limbs may be blocked and the blood is in a hypercoagulable state. Besides, AT III deficiency is prone to spontaneous thrombosis, which is a risk factor that further accelerates the occurrence of PE. At present, the detection of *SERPINC1* gene mutation is a necessary method for the diagnosis of AT III deficiency and the identification of the subtype.^[17]^ After we excluded acquired AT III deficiency, genetic testing should be routinely performed on cases with decreased AT III activity levels.

For the treatment of patients with AT III deficiency, the selection and dosage of drugs need to be considered from multiple aspects. AT III itself is a slow inhibitor of thrombin, factor Xa, and factor IXa. Only in the presence of heparin can it play a corresponding anticoagulant effect. In contrast, the decreased AT III activity will directly affect the anticoagulant effect of heparin, including low molecular weight heparin. For decades, vitamin K antagonists (such as warfarin) have been used for the treatment of VTE patients, including patients with AT III deficiency. Warfarin is an effective anticoagulant for patients with AT III deficiency and has been widely accepted.^[18]^ The patient reported in this article was treated with warfarin combined with enoxaparin sodium before INR reached the standard, but patients with AT III deficiency are usually accompanied by heparin resistance, so the anticoagulant effect of low molecular weight heparin at ordinary doses is poor, and higher doses of low molecular weight heparin (>35,000 U/d) are needed to achieve therapeutic activation of partial thromboplastin time. After INR reached the standard, warfarin 3 mg, once a day was given and maintained for a long time. After discharge, the patient was referred to the outpatient clinic regularly, and the computed tomography pulmonary angiography reexamination showed that the thrombus had been completely absorbed. For new oral anticoagulants, such as rivaroxaban and edoxaban, only a few cases have been reported in the treatment of patients with AT III deficiency,^[19,20]^ so the clinical evidence for the use of new oral anticoagulants in such patients is lacking. However, as direct inhibitors of factor Xa, which can exert anticoagulant effects independent of AT III, these drugs may play an important role in the treatment of acute venous thromboembolism and the secondary prevention of patients with AT III deficiency, and do not require regular monitoring of INR, or can be used as follow-up maintenance anticoagulant therapy for patients with AT III deficiency.

## 5. Conclusions

In summary, this article reports the data of a patient with PE caused by type I hereditary AT III deficiency due to the mutation of the *SERPINC1* gene encoding AT III. The patient had no typical clinical manifestations of PE at the onset and was hospitalized in our hospital for viral myocarditis. Due to significantly elevated D-dimer and venous thrombosis found in the right lower limb, PE was diagnosed after the completion of a chest imaging examination. At first, enoxaparin sodium combined with warfarin was given for anticoagulant therapy, and when INR reached the standard, warfarin was maintained for a long time, achieving good efficacy. Therefore, for this type of type I AT III deficiency combined with PE patients, warfarin is an effective anticoagulant drug. However, the use of warfarin requires regular monitoring of INR, which will reduce the compliance of patients after discharge. New oral anticoagulants may be used as follow-up maintenance anticoagulant therapy for patients with AT III deficiency, but this may need more clinical research support.

## Author contributions

**Conceptualization:** Miaoyuan Lin.

**Formal analysis:** Miaoyuan Lin, Xishi Sun.

**Writing—original draft:** Miaoyuan Lin.

**Supervision:** Xishi Sun, Jun Wu.

**Writing—review & editing:** Xishi Sun, Jun Wu.

**Methodology:** Jun Wu.

**Visualization:** Jun Wu.
